# Supplementary material for: unitas: the universal tool for annotation of small RNAs
Source: BMC Genomics. 2017 Aug 22;18:644. doi: 10.1186/s12864-017-4031-9 (PMC5567656; doi:10.1186/s12864-017-4031-9)
Supplement: Supplementary file 1 — Title of data: Supplementary Methods. Description of data: Describes how the artificial sncRNA datasets that were used for testing purposes were generated. (PDF 48 kb) [file 12864_2017_4031_MOESM1_ESM.pdf]

Supplementary Methods

Building test datasets for miRNA annotation

We used human mature miRNA sequences and miRNA precursor sequences from miRBase to build a miRNA test dataset comprising a specified number of unmodified and modified miRNAs. The modifications comprised all possible combinations of 5' and 3' offsets from -2 to +2 nucleotides and in addition up to 3 nucleotides 5' offset without 3' offset and vice versa. We further added A, AA, U or UU 3'-tailed versions for each canonical and offset miRNA. Finally, for every sequence we obtained this way, we added a counterpart comprising one internal sequence modification at position ten.

miRNA sequences were generated based on their precursor sequences, meaning that every precursor hairpin can yield the same number of miRNA reads, given that an offset modification not extends the resulting miRNA sequence beyond the precursor sequence. Based on the latter, and the fact that some miRNA genes have several (possibly slightly different) genomic copies, the obtained number of reads for different miRNAs varies (Additional file 8: Table S7). Both the test dataset and the Perl script that was used to create the test dataset are freely available at http://www.smallrnagroup.uni-mainz.de/data/UNITAS/resources.html.

Building test datasets for tRNA annotation

We used human tRNA sequences downloaded from Genomic tRNA database to build all types of tRNA fragments from each annotated tRNA sequence. 5'tRFs matched the 5' end of a tRNA and ranged in size from 18 to 22 nt. 3'tRFs matched the 3' end of a tRNA and ranged in size from 20 to 24 nt. Each tRNA was cut into two pieces at positions 32 to 36 to yield 5'-halves and 3'-halves. Miscellaneous tRNA fragments ranging from 18 to 40 nt in size were created using internal tRNA sequence starting from position 8. tRNA trailer sequences were used to generate 18 to 40 nt tRF1s. Both the test dataset and the Perl script that

was used to create the test dataset are freely available at http://www.smallrnagroup.uni-mainz.de/data/UNITAS/resources.html.

Building test datasets for phasiRNA annotation

In order to test the sensitivity and accuracy of phasiRNA prediction, we generated a collection of different artificial test datasets comprising those that contain solely phased RNAs, those that contain no phased RNAs and precisely defined mixtures of both. We first generated artificial phasiRNA datasets *in silico*, applying the following procedure: For each human chromosome including chromosomes X and Y, we quasi-randomly chose 91 loci that served as template for generation of phased small RNA sequences, starting at coordinate 1,000,001. If the 1050 bp downstream sequence did not comprise stretches of N, we generated 100 subsequences representing 50 artificial phased 21 nt RNAs per strand, directly adjacent to each other, with two nucleotides offset for plus strand sequences. For each next locus, we moved 10 kb downstream and generated siRNAs as described above. While keeping 100 artificial small RNAs for the first locus of a chromosome, we randomly rejected an increasing number of artificial siRNAs ending with 10 artificial small RNAs at locus 91 of a given chromosome. This procedure resulted in 125,125 artificial phased siRNAs representing 116,017 non-identical sequences.

To allow for quantification of false-negative as well as false-positive phasiRNA prediction, we prepared datasets containing our artificial phased small RNAs and an increasing number of non-phased sequence reads from human miRNA datasets representing Universal Human Reference RNA (Agilent Technologies, #750700) and human brain total RNA (Life Technologies, #AM6050) [1]. The two human miRNA datasets (SRA accessions: SRR950876 and SRR950878) were downloaded from NCBI's Sequence Read Archive. 3' adapter sequences from human miRNA datasets were clipped screening for $TGGAATTCTCGGN_x$-3' and only sequences ranging from 18 to 40 nt were chosen for further processing. For the different test datasets, we subsequently added 0, 1E+5, 5E+5, 1E+6, 2E+6, 3E+6, 4E+6 and 5E+6 sequence reads from SRR950876 (test datasets 1-8) or SRR950878 (test datasets 9-16) to the artificial phased small RNAs. We further generated

a second collection of test datasets just as described above, but assigning a sequence read count of ten to each artificial phased RNA (test datasets 17-32). We also used both miRNA datasets without adding artificial phased small RNAs to test for false positive phasiRNA prediction (test datasets 33 and 34, Additional file 6: Table S6). Test datasets 1-34 were mapped to the human genome GRCh38 with STAR (command line options: --outSAMstrandField All --outFilterScoreMinOverLread 0 --outFilterMatchNmin 15 --outFilterMatchNminOverLread 0 --outFilterMismatchNoverLmax 0 --alignIntronMax 1) [2], bowtie1 (command line options: -f -v 0 -k 10 -S -t) [3] and bowtie2 (command line options: -- local -p 16 -f -D 20 -R 3 -N 0 -L 8 -i S,1,0.50 -k 10 -t -x) [4], considering only perfect matches by subsequent filtering of SAM map files. The same aligners and settings were used to map sncRNA sequences from rice strains nipponbare and 93-11 [5] to the respective genomes [6, 7]. Test datasets 1-34 and the Perl script that was used to create artificial phased RNAs are freely available at http://www.smallrnagroup.uni-mainz.de/data/UNITAS/resources.html.

References

1. Mestdagh P, Hartmann N, Baeriswyl L, Andreasen D, Bernard N, Chen C, et al. Evaluation of quantitative miRNA expression platforms in the microRNA quality control (miRQC) study. Nat Methods. 2014;11:809-815.
2. Dobin A, Davis CA, Schlesinger F, Drenkow J, Zaleski C, Jha S et al. STAR: ultrafast universal RNA-seq aligner. Bioinformatics. 2013;29:15-21.
3. Langmead B, Trapnell C, Pop M, Salzberg SL. Ultrafast and memory-efficient alignment of short DNA sequences to the human genome. Genome Biol. 2009;10:R25.
4. Langmead B, Salzberg SL. Fast gapped-read alignment with Bowtie 2. Nat Methods. 2012;9:357-359.

5. Song X, Li P, Zhai J, Zhou M, Ma L, Liu B et al. Roles of DCL4 and DCL3b in rice phased small RNA biogenesis. Plant J. 2012;69:462-474.
6. Kawahara Y, de la Bastide M, Hamilton JP, Kanamori H, McCombie WR, Ouyang S, et al. Improvement of the Oryza sativa Nipponbare reference genome using next generation sequence and optical map data. Rice. 2013;6:4.
7. Rise Database. BGI Shenzhen, China. 2009. http://rise2.genomics.org.cn/page/rice/download.jsp. Accessed 10 Feb 2017.
